# A Roadmap for Participatory Chestnut Breeding for Nut Production in the Eastern United States

**DOI:** 10.3389/fpls.2021.735597

**Published:** 2022-01-03

**Authors:** Ronald S. Revord, Gregory Miller, Nicholas A. Meier, John Bryan Webber, Jeanne Romero-Severson, Michael A. Gold, Sarah T. Lovell

**Affiliations:** ^1^School of Natural Resources, Center for Agroforestry, University of Missouri, Columbia, MO, United States; ^2^Empire Chestnut Company, Carrollton, OH, United States; ^3^Department of Biological Sciences, 327 Galvin Life Sciences, University of Notre Dame, Notre Dame, IN, United States

**Keywords:** chestnut, *Castanea*, tree breeding, participatory, repository, conservation

## Abstract

Chestnut cultivation for nut production is increasing in the eastern half of the United States. Chinese chestnuts (*Castanea mollissima* Blume), or Chinese hybrids with European (*C. sativa* Mill.) and Japanese chestnuts (*C. crenata* Sieb. & Zucc.), are cultivated due to their high kernel quality, climatic adaptation, and disease resistance. Several hundred thousand pounds of high-quality fresh nuts are taken to market every fall, and several hundred additional orchards are entering bearing years. Grower-led on-farm improvement has largely facilitated this growth. A lack of significant investments in chestnut breeding in the region, paired with issues of graft incompatibility, has led many growers to cultivate seedlings of cultivars rather than grafted cultivars. After decades of evaluation, selection, and sharing of plant materials, growers have reached a threshold of improvement where commercial seedling orchards can be reliably established by planting offspring from elite selected parents. Growers recognize that if cooperation persists and university expertise and resources are enlisted, improvement can continue and accelerate. To this end, the University of Missouri Center for Agroforestry (UMCA) and chestnut growers throughout the eastern United States are partnering to formalize a participatory breeding program – the Chestnut Improvement Network. This partnership entails the UMCA providing an organizational structure and leadership to coordinate on-farm improvement, implement strategic crossing schemes, and integrate genetic tools. Chestnut growers offer structural capacity by cultivating seedling production orchards that provide financial support for the grower but also house segregating populations with improved individuals, *in situ* repositories, and selection trials, creating great value for the industry.

## Introduction

Chestnut (*Castanea* spp.) is currently a minor crop in the United States, and investment in chestnut breeding has been minimal to date (Anagnostakis, 2012). However, commercial production of Chinese chestnuts and complex hybrids is expanding in the eastern states, with over 300 bearing age orchards and an additional 300 newly established (1-5-year-old) orchards (USDA, 2018). Given an increasing chestnut grower base across diverse environments (Texas north to Nebraska and every state to the East), formalizing an organized breeding network would support and bolster this growth. Growers tend to be deeply passionate stewards and proponents of the crop, with a great desire to assist in and benefit from genetic improvement efforts. At present, most improvement efforts have been undertaken by growers, many of whom are members of the Northern Nut Growers Association (NNGA) and/or Chestnut Growers of America (CGA). However, improvement activities often occur independently and would benefit from greater organization and coordination. Researchers at the University of Missouri Center for Agroforestry (UMCA), who have worked on chestnut improvement for over two decades, are currently establishing a partnership with growers in the form of a participatory breeding network. This network aims to enhance and coordinate grower efforts by providing institutional support, including creating an online database of on-farm germplasm, robust genotyping, controlled crossing schemes, and dedicated, long-term research and selection activities.

We propose UMCA as an institutional home well-suited for this effort. The UMCA research farm [the Horticulture and Agroforestry Research Center (HARC)] maintains one of the largest repositories of chestnut cultivars in the United States. It is situated in the Missouri River Hills on very deep, well-drained Menfro silt loam soil, providing exceptional growing conditions for chestnuts. The UMCA research farm offers an excellent growing site and a secure and stable location to preserve genetic resources and display demonstration orchards. To support a robust, long-term breeding program, UMCA does have limitations: (i) finite land resources for growing and maintaining thousands of breeding progeny; (ii) periods of limited financial resources for long-term maintenance of the collections; and (iii) growing conditions that are different from those in many of the diverse growing regions of affiliated growers.

Consequently, since 2008, UMCA researchers and partner growers have widely disseminated half-sib offspring (pollinated by a highly diverse parentage) from the UMCA germplasm repository to growers throughout much of the eastern half of the United States. Approximately 7,000 offspring representing over 20 half-sib families of *C. mollissima* or complex hybrids cultivars are now of bearing age and are detailed by Miller (2016) and Revord et al. (2021). These plantings provide researchers and growers with robust, genetically diverse breeding populations from which growers are already identifying locally well-adapted, elite trees that serve their production needs and as candidate parents for future breeding efforts. As we enter the next cycle of genetic improvement, we envision much better coordination of growers’ efforts with institutional support to create a systematic but decentralized breeding program that serves the needs of various growers in various environments.

Chestnut as a food crop is in a unique position. Due to problems with clonal propagation, most growers in the eastern United States rely on seedling populations for commercial production. Thus, the industry needs a large number of genetically superior seedlings and this creates a built-in mechanism for large-scale genetic improvement, where desired traits are recombined into superior seedlings populations. Highly dedicated and connected growers often keep detailed records on seedling origin and the year-to-year performance of trees. The combination of engaged growers, dedicated researchers, and extant genetic resources offers a highly unique opportunity to develop a participatory chestnut breeding network coordinated through the UMCA.

## Chestnut – an Emerging Crop for the Eastern United States

The chestnut (*Castanea*), a member of the beech family (Fagaceae), has provided value to humans through timber products, ecosystem services, livestock feed, and edible nuts. Chestnuts have been used as a dietary staple for millennia (Anagnostakis, 2012). The genus *Castanea*, containing at least nine species, can be found on more than 2 million hectares in 25 countries (Beccaro et al., 2019). Chestnuts grow over a broad climatic range from subtropical to severe continental climates wherever rainfall and temperatures support deciduous forest. All chestnut species require well-drained acidic soils. Chestnuts are monoecious and obligate out-crossers. Pollen is spread primarily *via* wind with assistance from insects. All species are highly heterozygous, and within-species genetic variation is high. All chestnut species freely hybridize and form fertile offspring. Consequently, the genetic base for chestnut breeding is vast.

The Chinese (*C. mollissima*), European (*C. sativa*), and Japanese (*C. crenata*) species are most commonly grown for their edible nuts. Interspecific hybrids involving these species and other species, especially the American chestnut [*C. dentata* (Marshall) Borkh], are grown throughout the eastern United States. The most prevalent species by far is the Chinese chestnut owing to its superior nut qualities, climatic adaptation, and resistance to chestnut blight and phytophthora root rot. Today, several eastern states are each annually producing tens of thousands of pounds of chestnuts that meet the high-grade standards for commercial markets (USDA, 2018) and sell out within 2 months of harvest. A large demand continues to exceed the supply, even as mature orchards and plantings expand. In 2018, Iowa growers took 18,000 kg of high-grade chestnuts to market through the Prairie Grove Growers Association. Production capacity from 59 Missouri farmers ranges upwards from 4,500 kg. In Ohio, the Route 9 Cooperative growers have steadily increased production to 45,000 kg with five farms of bearing age. Similar trends are observed in half a dozen other states, soon bringing the number of bearing farms from 330 to over 600. Further, growth is ongoing as the UMCA distributed open-pollinated seed from its repository to over 90 growers in the Fall of 2020.

## Breeding and Selection Efforts

Chestnut’s economic success depends on consistent yields and high nut quality, both complex traits with multiple components. In general, Chinese chestnuts have desirable nut characteristics, including a round shape, desirable size (10 g or higher), easy peeling, and superior flavor and texture. Nut quality defects include small or flat nuts, bitter or astringent taste, low keeping quality, mealy texture, and blossom end rot caused by *Colletotrichum* spp. Defective cropping traits include nut drop within the bur, excessive crop load resulting in small nut size, light crop load, and year-to-year yield variability. The breeding goal for chestnut is to identify and combine desirable attributes into an elite group of superior parents with good combining ability while maintaining or expanding a broad genetic base to accommodate future genetic gain and respond to future problems and needs. Furthermore, adaptation to the changing climate is a major challenge, phenotypic plasticity for yield and nut quality under environmental extremes (excess moisture, drought, heat stress) can be addressed by evaluation in diverse environments. Thus, there is a need to systematically identify and utilize exceptional individuals within the expansive gene pool of on-farm germplasm.

Cultivar trials and germplasm repositories have not been prominent in the United States but have been done on a modest scale by private individuals and public institutions. Grafted cultivars can be maintained in modest quantities to support curation and trialing, but issues with delayed graft failure limit cultivation of grafted trees in commercial orchards. The Connecticut Agricultural Experiment Station, the University of Missouri, and Michigan State University have ongoing germplasm curation and evaluation efforts. The USDA recently removed its orchard in Byron, GA; however, scions and seeds were collected, grown, and preserved by private enthusiasts and UMCA.

Today, chestnuts (Chinese or Chinese hybrid seedlings) are grown over a wide geographic and climatic range in the United States. After decades of evaluation, selection, and sharing of plant materials, growers have reached a threshold of genetic improvement such that commercially viable seedling orchards can be reliably established simply by planting offspring from elite selected parents (i.e., exceptional on-farm selections that performed well in subsequent replicated testing, many which were subsequently named and added to The Connecticut Experimental Station cultivar database; Anagnostakis, 2019). Collectively, growers see the substantial progress made; they see the substantial genetic variation in their plantings; they realize the potential for substantial improvement yet to be made. Growers recognize that if they continue to cooperate, and if they enlist the expertise and resources of a university, genetic improvement can achieve a rate and efficiency not otherwise attainable. In other words, individual interests are best served by a collaborative approach. Further, the economic viability of chestnut production increases in proportion to genetic improvement. While the growers’ orchard trees provide financial support for the grower, those same trees can serve as *in situ* repositories, evaluation trials, and a source of elite parents for the next cycle of improvement, creating great value for the industry.

## Participatory Plant Breeding

The efficacy of participatory plant breeding efforts is well-documented for many domesticated crops throughout the world over the last 30 years. Participatory plant breeding is currently employed to develop major crops (wheat, maize, oilseeds) adapted to local or low-input environments (Vincourt and Pierre, 2018; Bocci et al., 2020; Van Frank et al., 2020). Additionally, participatory plant breeding has contributed to the improvement of historically underutilized crops, such as quinoa (Murphy et al., 2016). In participatory plant breeding, growers are directly involved in the breeding program’s decision-making, particularly in the selection of early breeding generations (Morris and Bellon, 2004; Vincourt and Pierre, 2018). The strengths and weaknesses of growers and plant breeders tend to be complementary. Growers are deeply aware of the unique challenges of their land and available market opportunities, offering practical expertise that could be translated into selection criteria. Growers also possess resources that can extend the footprint of a breeding program (Weltzien and Christinck, 2017). Cooperation with growers expands program capacity, broadens impact, and accelerates adoption compared to traditional, stand-alone, institutional breeding programs (Sperling et al., 2001; Ceccarelli and Grando, 2007; Hoffmann et al., 2007; Dawson et al., 2008). There is an incentive for programs to opt for a participatory approach when many grower preferences and target environments characterize their grower stakeholder base (Ceccarelli and Grando, 2007).

### Grower Engagement, Priority Setting

Of particular importance is farmers’ in-depth knowledge of their crops, encompassing performance in regional or local conditions, cultural practices, trends in emerging new pests and diseases, markets, and consumer preferences (e.g., nut size and flavor). Examples from grain crops illustrate growers’ nuanced understanding of component traits, like inflorescence patterns of high-yielding cultivars (Thapa et al., 2009). Selection criteria can differ between growers and breeders and among growers, and for good reasons – with different production, marketing, and use conditions across regions. Thapa et al. (2009) studied how grower selection criteria could help breeders select locally adapted wheat. They demonstrated frequent synergies between breeders’ quantitative and growers’ qualitative evaluations (although there were differences), validating farmers’ ability to choose superior individuals with qualitative approaches on-farm. Critical differences in assessments between breeders and growers often reflect breeders’ prioritization of agronomic performance. On the other hand, growers have an intrinsic multi-trait approach that considers local conditions and preferences. If criteria differences are well-described, both classes can effectively be integrated into selection procedures in the early generations of a breeding program (Vom Brocke et al., 2010; Burman et al., 2018).

Grower involvement in conducting evaluations and making selections ensures that activities accurately reflect their preferred criteria and that sufficient genetic diversity is maintained within pedigrees for their traits of interest (Ceccarelli, 1996; Dawson et al., 2008; Rauf et al., 2010). Decentralized grower evaluations create the ongoing benefit of grower feedback to refine selection criteria, breeding targets (Ceccarelli and Grando, 2007), and breeding parent selection per respective target environments. Although decentralized selection and participatory plant breeding are different concepts, they are often employed together in practice (Ceccarelli et al., 2001).

### Decentralized On-Farm Selection

Variation in grower environments is complex and multi-dimensional, especially as climate change accelerates. Fluctuations in climate make breeding new cultivars that perform over a large environmental gradient exceedingly difficult (Döring et al., 2011). Decentralized selection is typically employed when grower environments are characterized by heterogeneity in stressors, which is exacerbated by the changing climate (Ceccarelli and Grando, 2020). Since complex selection pressures of different environments are challenging to impose at a single location, evaluation directly within the different environments can be effective and efficient (Atlin et al., 2001; Dawson et al., 2008), which is even applicable for climate change-related traits (Ceccarelli et al., 2013). Decentralization is also most effective when utilizing genetically diverse, early generation material before heavy selection pressure reduces allelic diversity (Dawson et al., 2008). Thus, a decentralized breeding program leads to greater maintenance of allelic diversity than a centralized approach.

Beyond gains from major adaptations, regional yield and yield stability benefits can be expected from planting locally adapted plant materials, referred to as ‘the home field advantage’ (Ewing et al., 2019). Promising genotypes developed in conventional breeding programs are often defeated by unpredicted susceptibility to a particular stress within 10 years of release (Vincourt and Pierre, 2018). Promoting grower resilience and sovereignty by providing the grower agency in protecting and increasing genetic resources is a powerful platform that breeder/population geneticists can leverage to preserve, maintain, and maximize genetic resources. Since chestnut trees are long-lived perennials, trees in any given location will occasionally experience some set of rare weather extremes during their decades-long lifespans. Evaluation and selection of individuals over a several-year span may miss the response to one or more particular severe stress event(s). By evaluating the same families over an array of locations that experience different weather, families at one place or another will be exposed to weather extremes, thus exposing genetic vulnerabilities or tolerances. So, decentralized selection allows selection for local adaptation and also more reliably exposes potential genetic vulnerabilities.

In the case of chestnut in the eastern United States, an essential characteristic of using seedling orchards for commercial production is that orchards are initially planted at high density (2-4x the final density). The trees are then thinned as they get larger and begin to compete with each other. The initial high density provides early income for the grower and the opportunity to cull the poorer performing individuals, increasing the average performance of the remaining trees and the planting overall. Consequently, chestnut growers are accustomed to observing trait variation and selecting superior individuals, and the standard practices for developing a seedling production orchard meshes well with on-farm selection for the breeding program.

Furthermore, since many trees are to be removed from a production orchard, it is practical to incorporate families from which limited superior individuals are expected. In practice, a production orchard can have two types of families: 1) those with a high mean performance value but low variance and 2) those with a low mean value of performance but high variance (e.g., interspecific hybrids). Populations from the first group represent potential incremental improvements from their parents. In contrast, the second population group would require greater culling but contain unique recombinants representing a high rate of genetic gain.

## A Participatory Network for Chestnut Breeding in the United States

**Figure 1:**
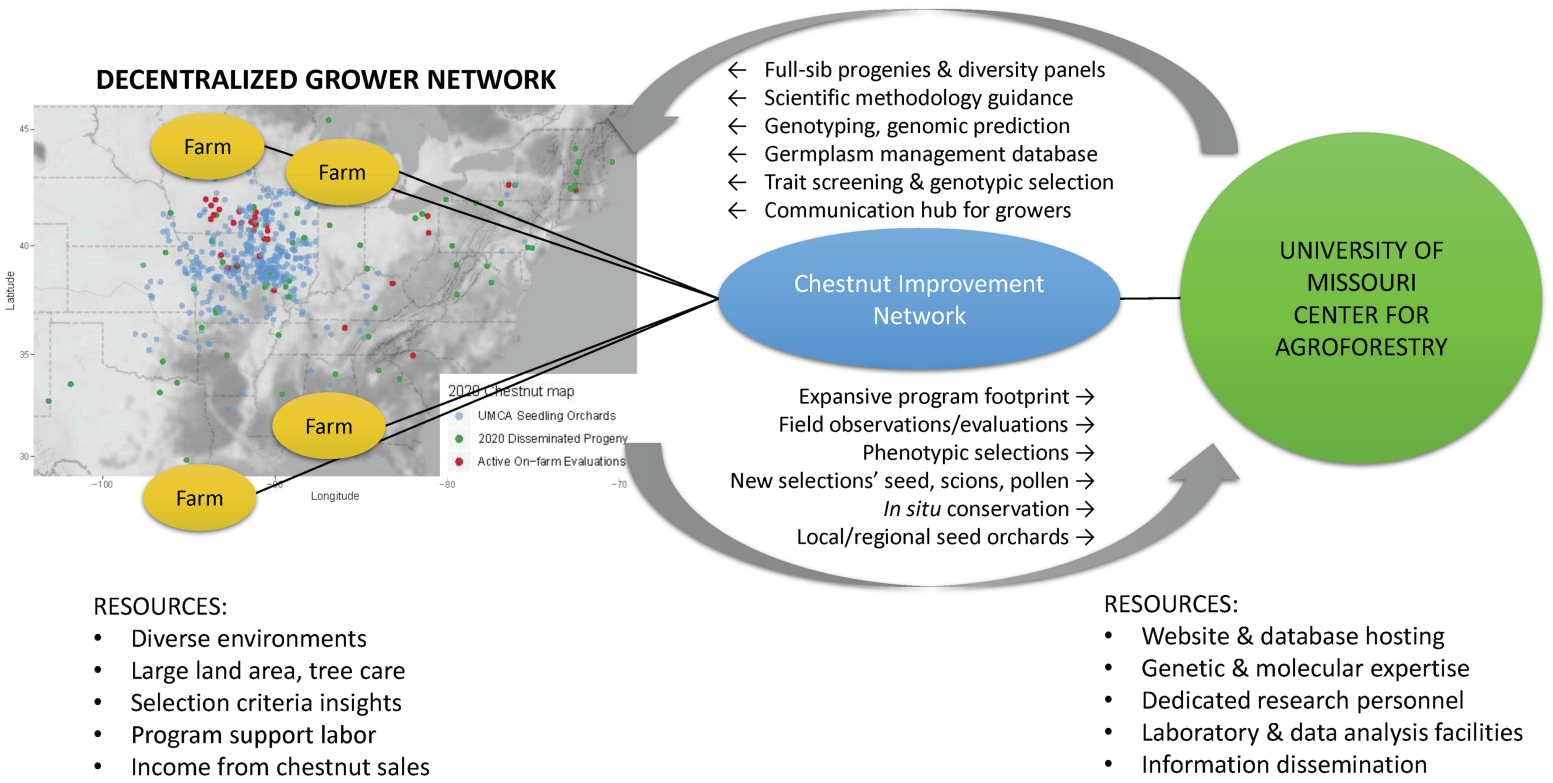
A diagram showing the complimentary roles, resources, and activities amongst the Chestnut Improvement Network. The map depicts the distribution of a grower subset that is cultivating seedling orchards from the University of Missouri Center for Agroforestry repository: bearing orchards (blue), 2020 seed distributed (green), and current on-farm evaluations (red).

The UMCA and chestnut growers throughout the eastern United States are partnering to form a participatory breeding program – the Chestnut Improvement Network. The UMCA is committed to providing an organizational structure and leadership in carrying out coordinated genetic improvement. Chestnut growers offer structural capacity by cultivating seedling production orchards that double as decentralized Chestnut Improvement Network breeding populations. Figure 1 depicts the complementary roles, resources, and activities between growers and the UMCA and; organized through the Chestnut Improvement Network.

### Germplasm

The UMCA initiated an extensive germplasm collection and evaluation effort (Mori et al., 2017) in the late 1990s with the help of key partners. The primary collection was assembled into a field repository between 1996 and 2005 and represented 65 cultivars conserved for long-term evaluation (Hunt et al., 2004). Fifty-four cultivars are still maintained and are described in Revord et al. (2021): 39 *C. mollissima*, six *C. sativa* × *C. crenata*, and nine other various or complex hybrids. Eleven cultivars, mostly hybrids (e.g., *C. sativa* × *C. crenata* ‘Colossal’), have been lost due to blight or poor growth. Based on geographic origin and descriptions from their donors, 12 of the collection’s most promising cultivars were selected for a replicated performance trial (established in 1999; Hunt et al., 2004, 2012). A foremost objective was to identify individuals well-adapted to the mid-Missouri climate with consistent production and nut qualities suitable for commercial markets (e.g., large, round, sweet, easy peeling, and low defects). These evaluations at UMCA and concurrent evaluations by growers have been used to inform seedling orchard establishment across the eastern chestnut region in the United States.

The distribution of seedlings from the UMCA repository and other sources has populated the chestnut growing region with genetically diverse half-sib progeny from *C. mollissima* or complex hybrid cultivars. Families and individuals have shown and will continue to show differential responses to abiotic and biotic stressors throughout the chestnut growing region. Winter cold hardiness and short growing seasons are major selection pressures in northern environments (USDA hardiness zones five and lower). Further, newly expanding shoots in the spring are vulnerable to temperatures below −2.2°C, so spring frost is a critical concern throughout the chestnut region. Thus, there is also a need to select for frost avoidance (i.e., late leafing). Pests and diseases, such as chestnut gall wasp, chestnut weevil, phytophthora root rot, chestnut blight, oak wilt, blossom end rot, and others, are all maladies for which genetic resistance exists and, in some cases, may be the only form of control available.

Together, growers and the UMCA are systematically evaluating on-farm progeny as potential parents of genetically diverse full- and half-sib populations to be tested under various local conditions. Selection pressure for local adaptation to various growing conditions is only effective when adequate genetic variation exists and selective conditions (e.g., severe cold) occur. Seedling orchards will also diverge for various economic traits, which are under assessment in tandem with adaptive characteristics. Consequently, the multi-trait selection requirements of on-farm evaluations mean they must be based on large populations, which become effectively larger when coordinated across many farms. Collective grower knowledge, insights, and ideas are also critically important so that evaluations can be continually refined to identify individual trees that best serve growers’ needs (Hunt et al., 2012).

**Figure 2:**
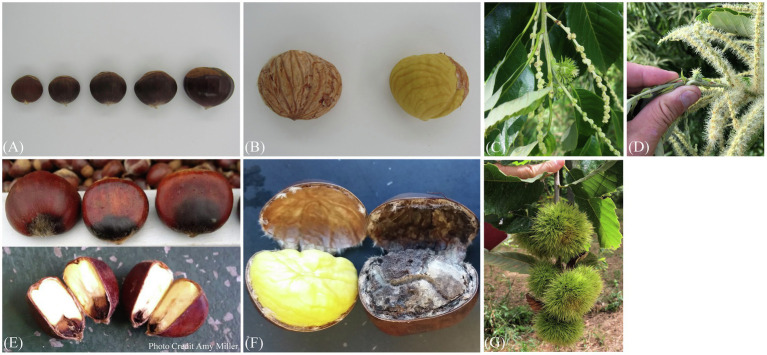
Select key traits of interest in chestnut improvement are displayed. **(A)** Nut size from left to right is 5.2, 8.5, 13.7, 17.9, and 26.7 g, respectively. Nut size above 10 g is preferential, and larger nut size is favored by certain markets. **(B)** Easy pellicle removal on the right vs. undesirable pellicle adherence on the left. **(C)** Male sterility is characterized by lack of stamen production. **(D)** Individuals with male sterility have approximately 2-fold higher yields than those that are male fertile. **(E)** Blossom end rot, caused by a fungus, *Colletotrichum gloeosporioides*. **(F)** Spoilage mold incidence during 60-day storage. **(G)** A desired five burs per shoot in Chinese chestnut (*Castanea mollissima*).

At present, over 600 selected seedling trees are under evaluation on-farm for seven agronomic traits, five leaf morphologies, 16 nut and kernel quality traits, and the incidence of seven pests and diseases (Table 1, Figure 2). The trees under evaluation represent over 40 growers and 20 states. UMCA specialists are carrying out assessments of agronomic traits and pest/disease incidence on-site (from 2020 to 2023). Leaf and nut samples are collected and brought to UMCA for later evaluation. Growers outside of our geographic range of in-person evaluations can participate by shipping 50-nut samples to the UMCA for evaluation. Field phenotyping guides are available to distant participants as an optional component of data collection. Multivariate analysis will explore the genetic diversity that exists for these traits amongst the on-farm selections. Additionally, genotyping with a set of around 60 EST-SSR markers (Romero-Severson, unpublished) will confirm maternal parentage, identify paternal parentage, ascertain the ancestry of complex *Castanea* hybrids, and provide permanent genetic fingerprints for the seedling trees. This marker set was derived from the *C. molissima* ‘Vanuxem’ reference genome (Staton et al., 2020) and subsequently cross-referenced in the to be published *C. dentata* reference genome. Growers collect dormant twigs in the winter or juvenile leaf tissue following bud break for DNA extraction and genotyping, regardless of their geographic location. The core collections assembled from this research will be conserved *via* combined *in situ* management in addition to the *ex situ* repository at HARC. Subsequently, the materials will be incorporated into improvement schemes as a part of the decentralized breeding program with chestnut growers.

**Table 1:** Descriptive standards for phenotypic data collected during on-farm and field trial observations.

Trait	Definition	Methodology	Reference
Production			
Bearing	The tree is producing fruit	The presence or absence of fruit on the tree	
Vigor	Overall size and shoot extension and growth relative to nearby trees of the same age	Visual rating: low, medium, or high	UPOV, 2017
Crop load	Overall estimate of the crop load based on the number of burs in the canopy compared to other trees of similar age in the orchard	Visual rating: none, low, medium, high, or extra high	Greg Miller, personal communication
Tree morphology			
Trunk diameter	Measurement of the size and growth of the tree at a given location	Calculated from the circumference of the tree (cm) taken at 30 cm above the root collar using a field tape	
Branch growth habit	The structure of the canopy of the tree	Visual rating: upright, semi-upright, spreading	UPOV, 2017
Degrees of looseness	The structure of the canopy in terms of light penetration	Visual rating: dense, intermediate, loose	Greg Miller, personal communication
Leaf morphology			
Leaf length	Measurement from proximal end to basal end of the leaf	mm	UPOV, 2017
Leaf width	Measurement from side to side at its widest point	mm	UPOV, 2017
Petiole length	Measurement of the petiole attached to the leaf	mm	UPOV, 2017
Leaf length/petiole ratio	Ratio of the leaf length and the petiole		UPOV, 2017
Leaf length/width ratio	Ratio of the leaf length and the petiole		UPOV, 2017
Leaf shape	Characterization of the leaf shape	Lanceolate, narrow elliptic, broad elliptic	UPOV, 2017
Leaf margin shape	Characterization of the margin of the leaf	Needle shape, acute, flare shape	UPOV, 2017
Underside pubescence and hair	Morphology of the underside of the leaf	Presence or absence of pubescence or hair on the underside of the leaf	
Nut and kernel morphology			
Nut width	Measurement from side to side of the nut	mm	Poljak et al., 2012, 2016
Nut length	Measurement from top to bottom of the nut	mm	Poljak et al., 2012, 2016
Nut depth	Measurement from front to back of the nut	mm	Poljak et al., 2012, 2016
Number of flat sides	Count of number of flat sides of the nut	0 (round), 1, 2	Poljak et al., 2012, 2016
Nut mass	Measurement of the mass of the nut	g	Poljak et al., 2012, 2016
Ease of peeling	Measurement of the peeling process	0 (peels in one or two chunks), 1 (peels in 3 or 4 chunk), 2 (difficult to peel and shell breaks into small pieces)	
Pellicle adhesion	Measurement of the pellicle on the kernel	0 (75 to 100% pellicle removal), 1 (50 to 75% pellicle removal), 2 (0 to 50% pellicle removal)	UPOV, 2017
Kernel invagination	Pellicle growing into the kernel	Presence or absence	UPOV, 2017
Nut embryony	Number of embryos forming the kernel	Mono- or poly- embryonic	UPOV, 2017
Kernel color	The color of the kernel under the pellicle	Yellow, light yellow, white	UPOV, 2017
Pests and disease			
Chestnut blight or other stem cankers	The formation of trunk/stem cankers on growing trees using a 5-level rating scale	0 = no cankers, 1 = few superficial cankers with no effect on tree growth, 2 = more obvious swollen cankers with deleterious effects on growth, but no stem death, 3 = obvious swollen cankers with some branch death and/or severe stunting of branch growth, perhaps epicormic branching, 4 = large branches dead with 30–70% of the crown dead, usually basal sprouting, 5 = whole tree dead from a canker with stump sprouting	Hebard, 2005
Phytophthora	Tree death without sprouting, phytophthora confirmed with diagnostic test or presence of dark “flaming” under the bark at base of the tree	Presence or absence	Santos et al., 2015
Oak wilt	Tree death or decline with oak wilt symptoms including leaf shedding	Presence or absence	Greg Miller, personal communication
Oak shot hole leaf miner	A very small fly that lays its eggs by piercing developing leaves with her ovipositor. The holes where eggs were laid become larger as the leaves expand.	0, 1, 2, or 3 scale based on severity	Boggs, 2021
Asian gall wasp	Formation of galls on the shoot tips	0, 1, 2, or 3 scale based on severity	Hunt et al., 2012
Chestnut weevil	Evidence on the outside or inside of the nut from chestnut weevil larvae	Presence or absence	Hunt et al., 2012
Chestnut blossom end rot	Black spoilage on the proximal end of the nut. Also referred to as chestnut anthracnose	Presence or absence	Miller, 2017
Tree phenology			
Green leaf tips visible	First green leaf tips just visible	Julian date	Larue et al., 2021
First leaves unfold	Leaves first beginning to unfold	Julian date	Larue et al., 2021
Beginning shoot growth	Beginning of shoot elongation	Julian date	Larue et al., 2021
First appearance of male catkins	First appearance of developing male catkins	Julian date	Larue et al., 2021
First appearance of female inflorescence on bi-sexual catkins	First appearance of female inflorescence on bi-sexual catkins	Julian date	Larue et al., 2021
First male flowers open	Male catkins first opening	Julian date	Larue et al., 2021
10–20% of male flowers open	10–20% of male catkins open	Julian date	Larue et al., 2021
Full male flowering	At least 50% of male catkins open	Julian date	Larue et al., 2021
Catkins fading	At least 50% of male catkins turning brown	Julian date	Larue et al., 2021
Stigma visible	Stigma of the central female inflorescence visible	Julian date	Larue et al., 2021
Full receptivity	Full receptivity of female inflorescence: stigma elongated and open	Julian date	Larue et al., 2021
Female flowers fading	At least 50% of female flowers have brown stigmas	Julian date	Larue et al., 2021

### Formalizing the Chestnut Improvement Network

The value of on-farm selections as a genetic resource comes from the whole of assembled collections rather than individual trees and locations. Thus, to maximize the benefit to participating growers, the selections from network farms must be made available to the grower network, with the caveat that local or regional adaptations may limit wide-range applicability. At this stage of chestnut industry development, growers are not in competition with each other. Instead, they are striving to attain a critical industrial mass that will foster industry-wide economies of scale.

While on-farm locations provide the structural capacity to cultivate, identify, and conserve new and improved genetic variants, the institutional home provides the knowledge base, perspective, and facilities to design and coordinate an improvement scheme. Looking forward, we will deploy three population types to growers: (1) grafted seed orchard populations, (2) diverse panels of seedling families that are balanced in size, and (3) full-siblings of new pedigree schemes derived from controlled crosses. Population types one and two are deployed, using the UMCA ‘Peach’ × ‘Qing’ × ‘Kohr’ seed orchard and the cultivar repository, respectively. Full-sibling offspring from UMCA pedigree schemes will be disseminated starting 2021, with a volume of five to ten thousand individuals annually. At present, these three populations are systematically utilizing the diversity present in the HARC repository and represent a major advance since past *ad hoc* open-pollinated seed dispersal. On-farm selections will be incorporated into these schemes over the next several years.

Evaluation of new seedling progeny descending from these population types will be conducted by both UMCA specialists and regional network technicians. A germplasm management database, E-Brida 6.0 (E-Brida, 2021), will facilitate quality control measures for accurate breeding stock record-keeping from deployment through data collection. QR code labels will ensure accurate nursery handling, field layout, and plot map documentation during on-farm establishment. These labels will be replaced as seedlings enter bearing years and data collection begins. E-Brida’s field application aids data collection quality control and ease by using tablets. Scanning the QR labels routes the user to the pedigree ID of the respective seedling. Data Properties (i.e., phenotypes) are predefined within the system and “toggled” on/off based on the assessment activities of the day/season to form digital data sheets with consistency across the network. Time-stamped images can also be uploaded in tandem with phenotypes to assist with validating data quality. Data on the application can be synced and archived on the cloud database once the tablet is connected to Wi-Fi.

Data collection priorities are hierarchical and vary by population type, family, and seedling age. Generally, evaluation and selection at the progeny-level will occur in seed orchard families or at the individual level in diverse family panels and full-sibling pedigrees. Evaluation of progeny in seed orchard families will prioritize valuable commercial traits, such as yield, interannual yield variability, nut quality, and defects. Mean progeny performance and variance will be assessed by replication (*n* ≥ 30 trees, *r* = 4–5) in complete randomized blocked orchards. Evaluation of individual trees with diverse family panels and full-sibling pedigrees incorporates wider criteria but on a family-specific basis respective to the goals of that pedigree/cross (e.g., narrow harvesting window, nuts that drop from burs, lower quality defects). Evaluation criteria will be expanded (e.g., to yield, nut attributes) on “retained” individuals that represent improvements for the requisite traits. Progeny tests will guide the commercial scaling of select seed orchards per environment, and individual selections will be incorporated into breeding schemes and replicated testing in ideal environments.

Replicated performance trials can subsequently serve as seed orchards and genetic repositories, as has happened, and will continue at the UMCA farm. Institutional involvement in the breeding program will allow more sophisticated phenotyping and genotyping, leading to more strategic crossing schemes, faster genetic gains, and integrity of germplasm tracking. Many US chestnut farmers have strong skills and experience in making selections from large populations of individuals. Growers are often capable and enthusiastic about carrying out field evaluations with proper training. UMCA specialists will continue to facilitate on-farm evaluations on a scale that matches the volume of next-generation seedling orchards planted.

The UMCA is committed to providing this institutional capacity, both in infrastructure and leadership, to the decentralized genetic improvement of chestnuts. We will work with growers to develop the organizational structure, record-keeping, and information dissemination to serve growers’ interests and our university mission. This approach can also be adapted to other long-lived perennial crops, as this is an effective way to conduct broad-scale genetic improvement programs for such crops.

## Data Availability Statement

The original contributions presented in the study are included in the article/**Supplementary Material**, further inquiries can be directed to the corresponding author.

## Author Contributions

RR, GM, NM, and JW drafted the manuscript. RR and GM edited the manuscript. GM, SL, and JR-S contributed significantly to conceiving, writing, and editing the manuscript. GM has been a pivotal leader in conceiving and organizing participatory chestnut improvement in the eastern United States for decades. GM and MG have curated cultivars and disseminated their seedlings to growers. RR, GM, JR-S, and MG initiated on-farm evaluations and the Chestnut Improvement Network in partnership with growers. All authors contributed to the article and approved the submitted version.

## Funding

This work is supported by the USDA Agricultural Marketing Service, Agreement No. AM190200XXXXG010, and the University of Missouri Center for Agroforestry and the USDA/ARS Dale Bumpers Small Farm Research Center, Agreement numbers 58-6020-6-001 and 58–6020-0-007 from the USDA Agricultural Research Service.

## Conflict of Interest

The authors declare that the research was conducted in the absence of any commercial or financial relationships that could be construed as a potential conflict of interest.

## Publisher’s Note

All claims expressed in this article are solely those of the authors and do not necessarily represent those of their affiliated organizations, or those of the publisher, the editors and the reviewers. Any product that may be evaluated in this article, or claim that may be made by its manufacturer, is not guaranteed or endorsed by the publisher.
